# Commentary: DCD liver transplant in patients with a MELD over 35

**DOI:** 10.3389/fimmu.2024.1404948

**Published:** 2024-07-11

**Authors:** Michelle C. Nguyen, Xingjie Li, Kunam S. Reddy, Amit K. Mathur

**Affiliations:** Department of Surgery, Division of Transplant Surgery, Mayo Clinic Arizona, Scottsdale, AZ, United States

**Keywords:** DCD liver transplantation, machine perfusion (MP), high-risk recipients, donor risk index, recipient outcomes

## Introduction

In 2022, a record 9,527 liver transplants (LTs) were performed in the United States, while a remarkable number of 12,862 additional candidates were added to the waiting list (1, 2). Despite the tremendous disparity between the number of waitlist patients and available organs, donation after circulatory death (DCD) livers remained underutilized compared to donation after brain death (DBD) livers. Only 11.3% of adult liver transplants in 2022 were performed with DCD livers (1, 2). The rates of “recovered but not transplanted” livers, often interpreted as discarded livers, were much higher in DCD compared to DBD (26.8% vs 7.2%) allografts. However, studies have shown that up to 50% of these discarded allografts may be suitable for transplantation (3, 4).

The rationale behind DCD underutilization stems from the concern that DCD allografts are more susceptible to ischemia-reperfusion injury and ischemic biliary complications impacting graft and patient survival, particularly in high-risk recipients (5). A previous cohort study found that DCD LTs in the subgroup of patients with higher MELD have worse graft survival compared to DBD LTs (6). However, with appropriate donor and recipient selection, acceptable outcomes can be achieved as demonstrated in the single-center study by Meier et al (7). The authors compared the outcomes of DCD LT for recipients with MELD ≥35 to a propensity-matched cohort of DBD-LTs. DCD organ acceptance was restricted to high-quality donors with age ≤40 years, graft steatosis ≤10-15%, and warm ischemia time (WIT) of ≤30 minutes. With these predetermined criteria, the 5-year patient survival for the DCD-LT cohort was comparable to DBD LT (85% vs 86%, p=0.843) (7). While noninferiority was demonstrated, only 41 DCD allografts were used compared to 1767 DBD allografts during a 30+ year period. Furthermore, we challenge the clinical implications of this conclusion in addressing the organ shortage given the current shift from static to dynamic liver preservation.

## Donor and recipient risk

Extrapolating from the author’s selection criteria, no UK DCD risk-defined “futile” livers were utilized. The DCD allografts that were transplanted would be stratified into low or high-risk groups, which would yield a predicted survival of >85% in selected recipients (8). Furthermore, these allografts were not considered in complex recipients such as those with portal vein thromboses or those requiring retransplantation. It is also important to note that waitlist mortality and dropouts during the study period were not insignificant as illustrated in Supplementary Figure 1 of the original manuscript. Considering that over 80% of candidates who die on the waitlist decline at least one marginal liver offer before death, granular data regarding the number of DCD offers declined for these recipients in the current study would be important in this context (9). Whether to accept a high-risk DCD liver or wait for a potential DBD offer is a difficult decision for transplant providers to make in the real-world clinical setting. In a national registry study, only 56.3% of MELD ≥35 recipients who previously declined a DCD offer ended up getting transplanted with a DBD allograft. The remaining 43% of those patients either died, dropped out, or were removed for other reasons (10). On the contrary, accepting a DCD liver, even from higher-risk donors, as evidenced by the subgroup analysis on DCD livers from donors age≥ 50, BMI≥30, and with a medical history of DM or HTN, lowers mortality risk compared to remaining on the waitlist, with the greatest survival benefit seen in the higher acuity recipients (10, 11).

## DCD liver transplantation in the era of machine perfusion

As the authors alluded to, improvements in organ procurement and preservation techniques will have implications in addressing the disparity between organ supply and demand while maintaining acceptable outcomes even in higher risk recipients. Organ perfusion strategies currently applied in the clinical setting include *in situ* normothermic regional perfusion (NRP), *ex-situ* hypothermic oxygenated perfusion (HOPE) or *ex-situ* normothermic machine perfusion (NMP). Compared to static cold storage (SCS), these modalities have all been shown in randomized clinical trials and cohort studies to reduce the incidence of early allograft dysfunction, post-reperfusion syndrome, and ischemic cholangiopathy in DCD-LT (12–17). Furthermore, all modalities provide a platform for viability assessment, allowing for safe transplantation of previously discarded allografts (16, 18, 19).

With amelioration of ischemia-reperfusion injury and its downstream effects, utilization of marginal allografts in high-risk recipients should be considered a viable option in the era of machine perfusion. In the Normothermic Machine Perfusion of the Liver to Enable the high-risk recipients (NAPLES) project, Hann et al. described the short-term outcomes of transplanting suboptimal grafts preserved on NMP into retransplant recipients. No differences were found in 6-month graft and patient survival compared to allografts preserved in SCS (20). Similar conclusions were drawn in another study of recipients age ≥65 years, whereby the NMP group exhibited significantly lower operative time and inpatient complications (21). In clinical practice, historically deemed high-risk or futile DCD allografts preserved on NMP have been utilized for high-risk recipients including those with complex abdominal surgical history, high grade portal vein thrombus, and MELD ≥35, but robust data have yet to be published.

## Discussion

Balancing donor and recipient risk while considering the limited availability of organs remains a complex challenge. The authors have demonstrated conservative efforts to improve utilization of DCD grafts for high-risk recipients. Efforts to expand the donor pool will continue to drive the growth of DCD-LT performed in the US, and innovative perfusion technology provides a safe platform for rapid expansion (22).

The highest available evidence has demonstrated with high level certainty that when compared to SCS, HOPE significantly reduces serious complications, retransplantation rates, and improves graft survival and both HOPE and NMP improves overall biliary complications and non-anastomotic biliary strictures (23–25). Most of the trials included in these meta-analyses included extended criteria-DBD or DCD grafts. Currently, no trials have focused on comparing machine perfusion to SCS in high-risk recipients, but ensuring equipoise in such a trial design would be difficult given the evidenced benefits of machine perfusion in liver transplantation. Despite that, differences in regional factors and center policy as well as logistical and financial barriers prevent widespread clinical adoption. Standardized and robust reporting of more granular transplant clinical data as well as collaboration among device companies, clinicians, and regulators will be essential. Addressing these issues will allow centers to learn from the existing experience, and successfully increase utilization of DCD and other marginal donor grafts, ultimately providing timely access to transplant and reduce the waitlist mortality for recipients.

## Author contributions

MN: Conceptualization, Investigation, Writing – original draft, Writing – review & editing. XL: Conceptualization, Investigation, Writing – original draft, Writing – review & editing. KR: Writing – review & editing. AM: Writing – review & editing.
